# The effect of thymoquinone coating on adhesive properties of polypropylene mesh

**DOI:** 10.1186/s12893-017-0239-5

**Published:** 2017-04-17

**Authors:** Oktay Aydin, Kuzey Aydinuraz, Fatih Agalar, I. Tayfun Sahiner, Canan Agalar, Cem Bayram, Emir Baki Denkbas, Pinar Atasoy

**Affiliations:** 1 Department of General Surgery, Kirikkale University Medical Faculty, Tahsin Duru Cad. No:14, Yenisehir, Yahsihan, 71450 Kirikkale, Turkey; 2 Department of Infectious Diseases and Clinical Microbiology, Kirikkale University Medical Faculty, Tahsin Duru Cad. No:14, Yenisehir, Yahsihan 71450 Kirikkale, Turkey; 3 Department of Pathology, Kirikkale University Medical Faculty, Tahsin Duru Cad. No:14, Yenisehir, Yahsihan, 71450 Kirikkale, Turkey; 4 Advanced Technologies, Application and Research Center, Hacettepe University, Beytepe, Ankara, Turkey; 5 Biochemistry Division, Department of Chemistry, Faculty of Science, Hacettepe University, Beytepe, Ankara, Turkey; 6 Anadolu Medical Center in affiliation with Johns Hopkins Medicine, Cumhuriyet Mahallesi, 2255 sokak No:3 Gebze, 41400 Kocaeli, Turkey; 7 Department of General Surgery, Hitit Universiy Medical Faculty, Bahçelievler Mah. Çamlık Cad. No: 2, 19030 Corum, Turkey; 8 Department of Infectious Diseases and Clinical Microbiology, Fatih Sultan Mehmet Training and Research Hospital, İçerenköy, Ataşehir, 34752 Istanbul, Turkey

**Keywords:** Thymoquinone, Intraabdominal adhesion, Polypropylene mesh, Incisional hernia

## Abstract

**Background:**

An incisional hernia is a common complication following abdominal surgery. Polypropylene mesh is frequently used in the repair of such defects and has nearly become the standard surgical treatment modality. Though they are very effective in reducing recurrence, mesh materials exhibit a strong stimulating effect for intraabdominal adhesion. The thymoquinone (TQ) extracted from Nigella sativa seeds has potential medical properties. TQ has anti-inflammatory, antioxidant and antibacterial properties. The aim of this study is to coat polypropylene mesh with TQ in order to investigate the effect of surface modification on intraabdominal adhesions.

**Methods:**

TQ-coated polypropylene mesh material was tested for cytotoxicity, contact angle, surface spectroscopy, TQ content, sterility, and electron microscopic surface properties. An experimental incisional hernia model was created in study groups, each consisting of 12 female Wistar rats. The defect was closed with uncoated mesh in control group, with polylactic acid (PLA) coated mesh and PLA-TQ coated mesh in study groups. Adhesion scores and histopathologic properties were evaluated after sacrifice on postoperative 21th day.

**Results:**

Granuloma formation, lymphocyte and polymorphonuclear leukocyte infiltration, histiocyte fibroblast and giant cell formation, capillary infiltration, collagen content were significantly reduced in the PLA-TQ coated mesh group (*p* < 0.05). Though not statistically significant, likely due to the limited number of study animals, adhesion formation was also reduced in the PLA-TQ coated mesh group (p: 0.067).

**Conclusion:**

TQ coated mesh is shown to reduce adhesion formation and TQ is a promising coating material for mesh surface modification.

**Electronic supplementary material:**

The online version of this article (doi:10.1186/s12893-017-0239-5) contains supplementary material, which is available to authorized users.

## Background

Incisional hernias are a frequently encountered problem in general surgery. Up to 20% of standard abdominal surgeries end up in incisional hernia [1, 2]. Incidence of incisional hernia may even reach 70% in high risk patients and complicated cases [3]. Obesity, surgical site contamination and infection, concomitant surgical procedures, damage control surgery, bariatric surgery, poor surgical technique and previous unsuccessful repair of an incisional hernia increase the risk of incisional hernia [1, 3, 4].

Prosthetic mesh augmentation has become the standard surgical approach for incisional hernia repairs. There has been a significant decrease in hernia recurrence with the incorporation of prosthetic mesh in open abdominal wall repairs for ventral hernia [5–7]. Polypropylene mesh is the most commonly used prosthetic material in hernia repair. Although polypropylene mesh successfully reduces the recurrence rate of incisional hernias, it cannot be placed intraperitoneally for the risk of adhesion formation, intestinal erosions and entero-cutaneous fistula formation [8]. Therefore, many studies concerning surface, structure and material modification of polypropylene graft have been carried out resulting in composite mesh [9–11].

Reduction of polypropylene content of the mesh has been one of the first solutions. The loose knitted lightweight macroporous mesh materials had reduced adhesion scores due the decrease in both contact area and material while showing a good strength and lesser shrinkage [10, 11].

Adding an adhesion barrier layer or coating to the visceral surface of the polypropylene mesh has been another solution. Expanded PTFE has created a nonbiodegradable barrier between polypropylene mesh and the viscera. A biodegradable hydrogel layer of sodium hyaluronate, carboxymethylcellulose and polyethylene glycol coating, omega-3 fatty acid gel coating, titanium coating, polydioxanone and oxidized regenerated cellulose of polyglecaprone-25 coating have also reduced adhesions [12–15].

Peritoneal damage generates localized hypoxia and inflammatory response. Activation of procoagulatory and antifibrinolytic reactions by acute inflammation, increased lactate, reactive oxygen species and nitrogen species lead to increased intraperitoneal adhesion [16–18]. Increased intraperitoneal leukotriene B4 and prostaglandin E2 inhibit both the activation of plasminogen and fibrinolysis. Lactate is also increased and it strongly stimulates collagen synthesis and angiogenesis both of which increase adhesion formation [18]. The hypoxic human peritoneal fibroblast produces increased amounts of ROS and RNS. Nitric oxide is also shown to enhance collagen synthesis and angiogenesis. Administration of ROS and RNS scavengers has decreased the rate of intraabdominal adhesions [16–18].

Thymoquinone (2-isopropyl-5-methlybenzo-1, 4 quinone) (TQ) derived from Nigella sativa, a plant from the Ranunculaceae family, has been reported to have strong anti-inflammatory, antioxidant properties. TQ is a potent inhibitor of leukotriene B4 and thromboxane B2. It inhibits arachidonic acid metabolism in peritoneal leukocytes via cyclo-oxygenase (COX) and 5-lipooxygenase pathways. TQ also has scavenging activity against superoxide anion, hydroxyl radical and singlet molecular oxygen [19–22]. Modulation of NF-κB and TNF-α attenuates proinflammatory responses and oxidative responses [20].

In this experimental study, the effect of TQ coating of polypropylene mesh to reduce adhesion formation is studied in an experimental incisional hernia model. It was hypothesized that coating polypropylene with TQ would reduce the adhesion to the mesh due to the anti-inflammatory properties of TQ.

## Methods

### Study groups

Thirty-six female, Wistar albino rats, each weighing 260–280 gr, were used in the study. All the animals were kept at 30–70% humidity and under 12 h of daylight at room temperature for 1 week in the laboratory before the experiment was conducted. They were fed with standard rat chow and water ad libitum.

Control group (12 rats): Uncoated polypropylene mesh was used to repair the experimentally created abdominal fascial defect.

PLA group (12 rats): Polypropylene mesh coated with PLA was used to repair the experimentally created abdominal fascial defect.

PLA + TQ group (12 rats): Polypropylene mesh coated with PLA and TQ was used to repair the experimentally created abdominal fascial defect.

### Preparation of polypropylene meshes

Polypropylene mesh (Promesh® T, Surgical IOC, Rue Marengo, Saint Etienne, France) was used in the study. It is a heavyweight mesh with microporous structure (surfacic mass:97 ± 5 g/m^2^, pore size 60–80 μ). By using heavyweight microporous mesh which is known to induce immediate and heavy inflammation, we aimed to increase the power for detection of the proposed reduction in inflammatory response.

The mesh was cut into 25 mm x 25 mm squares. The samples were coated with PLA (as carrier), PLA and TQ (Thymoquinone, 2-Isopropyl-5-methyl-1,4-benzoquinone, %99, CAS Registry Number 490-91-5, purity ≥ 98%, molecular weight 164.20, molecular formula C_10_H_12_O_2,_ Santa Cruz Biotechnology, California, USA).

A PLA solution of 240 mg/ml concentration and a TQ + PLA solution with a TQ concentration of 100 mg/ml were prepared with methylene chloride as the solvent. The precut mesh materials were submerged in the solutions for 1 min. They were then left to air dry in dust-free, closed petri dishes and sterilized by ethylene oxide.

### Indirect cytotoxicity tests

Cytotoxicity tests were conducted in accordance with ISO 10993–5 standard using single layer murine fibroblast L929 cell line (Foot and Mouth Disease Institute, Ankara, Turkey).

#### Preparation of cell culture

The cell line was cultured in DMEM (Sigma-Aldrich, USA) culture medium enriched with 10% FBS (Biochrom, Germany). One percent L-glutamine (Invitrogen, USA) and 100 units/ml penicillin-streptomycin (Invitrogen, USA) were added to the culture. The culture media, which was held at 37 °C and 5% CO_2,_ was renewed every 2 days. When the cell population reached 80%, the cells were retrieved by 0.25% trypsin containing 1 mM EDTA solution (Invitrogen, USA), were counted by hemocytometer, and were kept to be used in indirect cytotoxicity tests.

#### MTT test and cytotoxicity

The MTT (3-(4,5-dimethylthiasole-2-il)-2,5-diphenyltetrazolium bromide) test was used to determine indirect cytotoxicity [23]. The mitochondria in the living cell disintegrates the tetrazolium ring of MTT dye changing the color from pale yellow to deep blue-purple. Group samples were incubated at 37 °C for 72 h in cell culture media. Two hundred μl of this media was then reacted with cells that were cultivated in a concentration of 2x10^5^ cells/ml in 96-well cell culture pits one night prior and 25 μl MTT dye was added. The cells were incubated for 4 h in darkness and were then broken down using isopropanol HCl. The blue formazan crystals in the mitochondria of the living cells were dissolved and analyzed with ultraviolet spectroscopy.

The cytotoxicity of mesh samples was assessed by calculating the relative cell viabilities amongst the sample groups with respect to the negative control.

$$ \mathrm{Cell}\ \mathrm{viability}\ \left(\%\right)=\left[\mathrm{Abs}570\left(\mathrm{sample}\right)/\mathrm{Abs}570\left(\mathrm{control}\right)\right]\times 100 $$

Abs570: Absorbance values read at the 570 nm wavelength.

Abs570(sample): absorbance value of cells that reacted with the samples.

Abs570(control): absorbance value of cells that did not react with the samples.

### Hydrophobicity

The stagnant drop contact angle was measured to assess hydrophilicity of the mesh. Eight random measurements were made for each study group using a Contact Angle Analyzer (Krüss, DSA 100, Germany).

### Chemical analysis of mesh samples

The graft material used in the study was chemically analyzed using Energy Dispersing X-Ray Spectroscopy (EDX) (Quanta 200 FEG, FEI Instruments, USA).

### Determination of the amount of TQ coating

The amount of TQ on the coating of mesh materials was determined by ultraviolet spectroscopy (UV Mini 1240, Shimadzu, Japan). TQ coated polypropylene mesh samples were immersed in 37 °C phosphate buffer of pH 7.4 for 3 days. Absorbance of buffer solutions was measured by ultraviolet visible zone spectrophotometry. The results were inserted in the equation of graph that was acquired by the calibration curve for known concentrations.

### Examination of polypropylene mesh surface

Mesh samples were examined by scanning electron microscopy (JEOL JSM-5600 Scanning Electron Microscope Model No: SIRIUS 10/7.5). The mesh samples were coated with approximately 55–60 Å pure gold and surface properties were examined under x50, x100 and x5000 magnification and 20 kV acceleration voltage.

### Sterility tests

Microbial cultures were taken before laparotomy and at relaparotomy on the 21st day to rule out intraabdominal infection.

### Surgical intervention

General anesthesia was induced in all animals by intraperitoneal administration of ketamine 90 mg/kg (Ketalar®, 500 mg/10 ml, Pfizer, USA) and xylazine 10 mg/kg (Rompun®, Bayer, Leverkusen, Germany).

A 4 cm midline laparotomy was accomplished in all rats under sterile conditions. A 25x25 mm defect of fascial and muscular layers was created in the anterior abdominal wall. The defect was covered with mesh in such a way that the whole surface of the mesh was exposed to the intestines resembling the contact of intestines with the mesh in intraperitoneal onlay mesh repair. The mesh was secured en bloc to peritoneum, muscle and fascia by interrupted 4/0 polypropylene sutures on all four sides of the mesh. The defect in the anterior abdominal wall was closed with uncoated polypropylene mesh in the control group, PLA-coated mesh in the PLA group and PLA-TQ coated mesh in the PLA-TQ group. The skin was closed by interrupted 4/0 polypropylene sutures (Prolene, Ethicon®, Johnson & Johnson, USA) [24].

No rats were sacrificed before the end of the study period. On the 21st postoperative day, rats were anesthetized by intraperitoneal application of ketamine and xylazine. The anterior abdominal wall was resected as a U-shaped flap containing the mesh. The rats were then sacrificed using a high dose ketamine. Microbial cultures were taken synchronously from each rat.

### Evaluation of adhesions

During relaparotomy, adhesions in all groups were evaluated by a blinded study participant using the Modified Diamond Scale [25]. Then, the U-flap was removed from the abdominal wall and placed in 10% formalin for microscopic evaluation.

### Histopathologic examination

Tissue samples were examined histopathologically by a pathologist who did not have any information about the specimen groups under light microscopy with x10-40 magnification after staining with hematoxylin - eosin. Granuloma formation, lymphocyte infiltration, PMNL infiltration, histiocytes, giant cells, capillary proliferation, collagen content, and fibroblast proliferation were studied.

### Exclusion criteria

Rats that fell ill or died during follow-up were excluded from the study and replaced with new ones.

### Statistical analysis

Kruskal-Wallis test was used for intergroup differences and the Mann–Whitney *U*-test was used for differences within each group (SPSS for Windows 17.0, SPSS Inc. Chicago, IL, USA). Minimum-maximum, median, and standard deviation were used for histopathologic examination. *p* < 0.05 was accepted as statistically significant.

## Results

### Cytotoxicity

There was no cytotoxic effect seen on the cells that interacted with uncoated mesh, whereas a reduction of 3 and 9% was observed in PLA and PLA-TQ group respectively, which may suggest a very slight cytotoxicity for the PLA-TQ group.

### Hydrophobicity

**Fig. 1:**
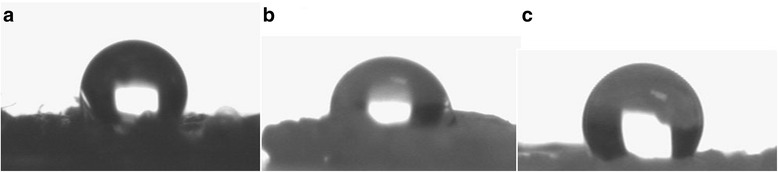
Stagnant Drop Contact Angle Measurement images of uncoated **a**, PLA coated **b** and PLA-TQ coated **c** mesh

As polypropylene mesh does not have hydrophilic groups on its surface, the water drop contact angle measurement was measured as 105.8° for uncoated mesh, 89.8° for PLA coated mesh and 94.7° for PLA-TQ coated mesh. The hydrophobicity was conserved after coating with PLA and PLA-TQ (Fig. 1).

### Analysis of mesh samples

The surface of the uncoated mesh exhibited only carbon atoms prior to coating, while the surface of the PLA coated and PLA-TQ coated mesh showed that there were compounds containing oxygen on the surface. Active TQ concentration per each graft piece was 245 ± 10 μg.

### Mesh surface

**Fig. 2:**
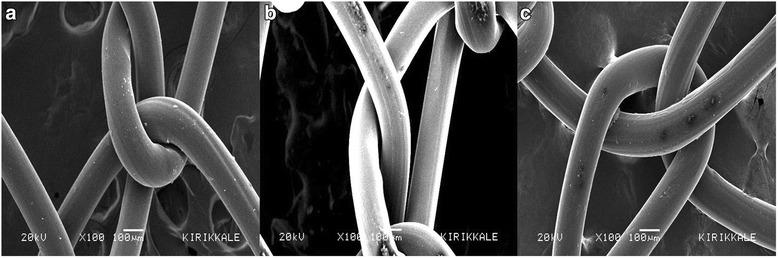
SEM images of uncoated **a**, PLA coated **b** and PLA-TQ coated **c** mesh (x100)

**Fig. 3:**
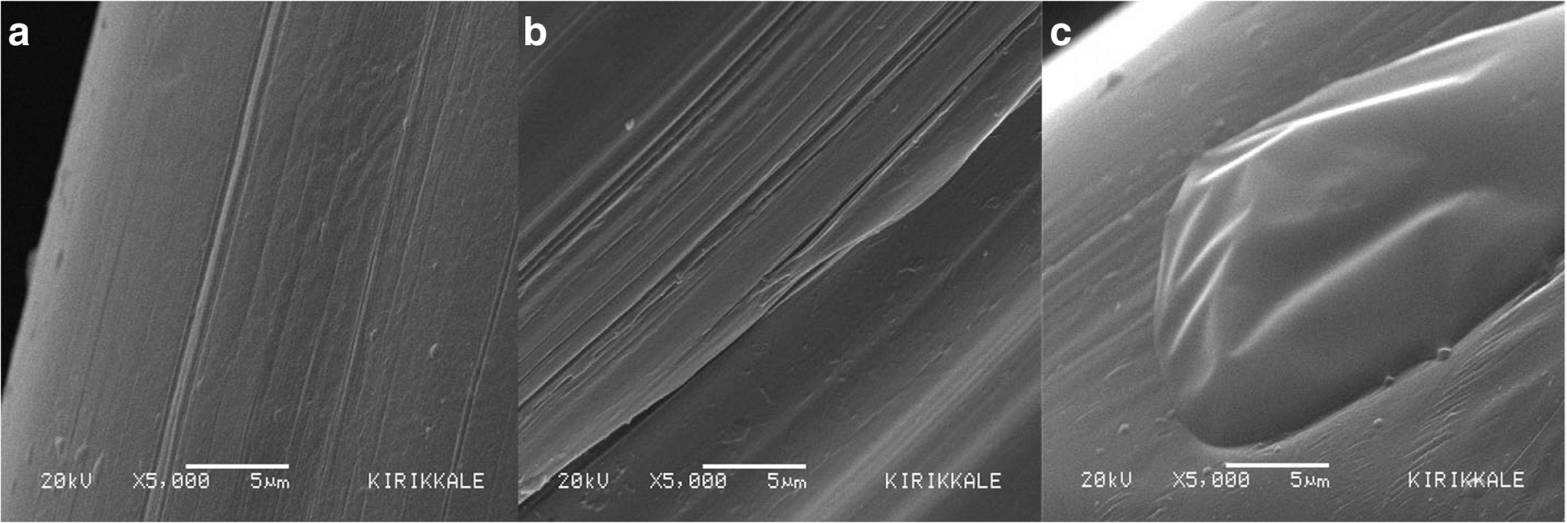
SEM images of uncoated **a**, PLA coated **b** and PLA-TQ coated **c** mesh (x5000)

Scanning electron microscopy under x50, x100 and x5000 magnification showed that surface patterns of the graft materials were smooth and coatings were homogenous without any bacteria or foreign material (Figs. 2 and 3).

### Sterility tests

No bacterial growth could be detected in cultures.

### Intraabdominal adhesion scores

Although there was a significant visual reduction for intraabdominal adhesions in the PLA-TQ group; it was not statistically significant (p: 0.067) (Table 1), (TQ coated PP mesh and adhesion-Additional file 1).

**Table 1:** Comparison of histopathologic results and adhesion scores

	Control (*n* = 12) (Mean ± SD)	PLA (*n* = 12) (Mean ± SD	PLA-TQ (*n* = 12) Mean ± SD
Granuloma formation	23,00±,522	18,50±,452	14,00±,000*
Lymphocyte infiltration	25,13±,515	20,63±,515	9,75±,718*
PMNL infiltration	26,67±,577	20,50±,492	8,33±,718*
Histiocytes	26,88±,452	17,71±,888	10,92±,515*
Giant cell formation	24,50±,389	21,50±,492	9,50±,000*
Capillary proliferation	23,17±,492	22,00±,622	10,33±,515*
Collagen content	22,92±,577	25,00±,492	7,58±,577*
Fibroblast formation	24,13±,452	22,96±,577	8,42±,603*
Modified Diamond adhesion score	20,75±,778	21,63±,669	13,13±,888**

*p = 0.00, **p = 0.067

### Histopathological results

Granuloma formation, lymphocyte infiltration, PMNL infiltration, histiocyte formation, giant cell formation, capillary infiltration, collagen formation, and fibroblast formation were significantly reduced in the PLA-TQ group when compared to control group (*p* < 0.05). There was a statistically significant difference for microscopic parameters for all groups (*p* < 0.005) (Table 1), (TQ coated PP mesh and adhesion-Additional file 1).

## Discussion

In this pilot study, we found no difference between contact angle measurements of naïve, PLA coated and PLA-TQ coated mesh. The coatings did not change the contact angle of the graft material suggesting that there will be no change in the physicochemical interaction of the coated graft materials. This strongly implies that the reduction in adhesion formation is not due to any change in physicochemical property of the mesh. In addition, the coatings also have not changed the texture of the surface of the graft material, which is evident in the SEM images.

Adhesion formation depends also on the patient and the wound. Adhesion is the answer of the peritoneum to the surgical insult magnified by the presence of a foreign body as it is in the case of mesh repairs. Peritoneal trauma creates an acute inflammatory response and localized hypoxia resulting in aggravated procoagulatory and antifibrinolytic activity [17, 18]. Therefore, controlling the extent of acute inflammation after surgery becomes more important in order to minimize peritoneal adhesions. TQ decreases TNF-α and IL-1β and PGE2, all of which contribute to the decrease in inflammation. TQ also inhibits LPS induced NF-κB by decreasing the upstream molecules of NF-κB, namely PI3K and Akt phosphorylation in microglial cells [26]. In our study, inflammation was significantly decreased in TQ coated mesh group consistent with anti-inflammatory effect of TQ.

The normal peritoneum has an intrinsic fibrinolytic activity [16, 18]. When the peritoneum is injured and mesothelial layer is exposed during surgery, inflammatory cells infiltrate the wound secreting proinflammatory cytokines. Activation of coagulation and complement cascades forms a fibrin-rich, serousanginous exudate. When it is cleared out properly and timely during by the fibrinolytic process, this exudate serves as a cornerstone for wound repair. But if this exudate is not dissolved timely, it acts as a matrix for fibroblasts becoming vascularized. Collagen deposition is increased in the adhesions resulting is fibrous permanent adhesions [18].

Localized hypoxia in the wound initiates intracellular responses under which the normal peritoneal fibroblast changes its phenotype irreversibly acquiring the adhesion phenotype. Hypoxia inducible factors activate anaerobic glycolysis. The net result is an increase in lactate which strongly stimulates collagen formation and angiogenesis, both of which are crucial for permanent adhesion formation [17, 18].

Human peritoneal fibroblast also produces increased amounts of ROS and RNS under hypoxia. Nitric oxide is shown to enhance collagen synthesis and angiogenesis. Administration of ROS and RNS scavengers has decreased the rate of intraabdominal adhesions [17]. In an experimental CCl_4_ induced hepatotoxicity model in mice, TQ has been shown to protect hepatocytes due to its antioxidant effect [20, 27]. TQ inhibits iron-dependent lipid peroxidation and increases catalase, superoxide dismutase, glutathione transferase and quinone reductase. TQ also acts as a radical scavenger and blocks this pathway of adhesion formation [17, 20]. In a recent experimental study by Bozdag et al., intraperitoneal administration of TQ has reduced peritoneal adhesions significantly. The grade of inflammation, hydroxyproline content were also significantly lower in the TQ administered group. These findings were correlated with the total antioxidant capacity of the samples which were significantly higher in the TQ administered group showing that the antioxidant effect of TQ is also responsible for the reduction in peritoneal adhesion scores in the TQ group [28].

Though insignificant (p:0.067), the decrease in macroscopic adhesion scores in our study supports the anti-inflammatory and antioxidant properties of TQ and are consistent with the results of the study by Bozdag et al. Timing for the evaluation of peritoneal adhesions, the nonparametric nature of adhesion score evaluation and the number of animals in study groups might explain why the reduction in adhesion scores was evident but not statistically different in TQ-PLA group when compared to other groups.

Active TQ concentration per each graft piece was 245 ± 10 μg in our study. The LD_50_ of TQ for intraperitoneal is to be 90.3 mg/kg. Doses up to 12.5 mg/kg can be given intraperitoneally without decreasing antioxidant properties [20, 27]. PLA and PLA-TQ have both proven not to be cytotoxic and the amount of TQ coating is well under toxic levels.

The reduction in inflammatory response induced by TQ coating of the mesh might recur to the mind that while reducing adhesion formation, TQ coating might also impair the strength of incorporation of the mesh into the peritoneal wall. In this pilot study, we aimed to evaluate the effect of low dose TQ in such a dose that would inhibit adhesion formation without being toxic. Therefore, tensile strength of the U-flap was not evaluated in this study. Measuring the strength of the repair in further studies will document if TQ can decrease adhesion formation without impairing wound healing and mesh incorporation.

## Conclusion

Inflammation and oxidative stress are the main reasons for adhesion formation. Coating polypropylene mesh with TQ which has potent anti-inflammatory and antioxidant activity is promising for the generation of a new composite graft that can be used intraperitoneally. Further experimental studies comparing the adhesion reducing effect of TQ coating with different anti adhesive mesh materials used in daily surgical practice will fortify this finding and may enable clinical application of TQ coated meshes.

## Additional file

Additional file 1: TQ coated PP mesh and adhesion. (DOCX 54 kb)

## Abbreviations

TQ: Thymoquinone
PLA: Polylactic acid group
MTT: 3-(4,5-dimethylthiasole-2-il)-2,5-diphenyltetrazolium bromide
