# Supplementary material for: The effect of thymoquinone coating on adhesive properties of polypropylene mesh
Source: BMC Surg. 2017 Apr 17;17:40. doi: 10.1186/s12893-017-0239-5 (PMC5393001; doi:10.1186/s12893-017-0239-5)
Supplement: Additional file 1: — TQ coated PP mesh and adhesion. (DOCX 54 kb) [file 12893_2017_239_MOESM1_ESM.docx]

**Intraabdominal Adhesion Scores**

| GROUP | Subject | Modified Diamond Adhesion Score | | | | |
| --- | --- | --- | --- | --- | --- | --- |
|  |  | Score-0 | Score-1 | Score-2 | Score-3 | Score-4 |
| CONTROL | C-1 |  |  |  | X |  |
|  | C-2 |  |  |  |  | X |
|  | C-3 |  |  |  |  | X |
|  | C-4 |  |  | X |  |  |
|  | C-5 |  |  |  |  | X |
|  | C-6 |  |  |  |  | X |
|  | C-7 |  |  |  |  | X |
|  | C-8 |  |  |  | X |  |
|  | C-9 |  |  |  | X |  |
|  | C-10 |  |  |  |  | X |
|  | C-11 |  |  |  | X |  |
|  | C-12 |  |  | X |  |  |
| PLA | PLA-1 |  |  |  | X |  |
|  | PLA-2 |  |  |  |  | X |
|  | PLA-3 |  |  |  |  | X |
|  | PLA-4 |  |  |  | X |  |
|  | PLA-5 |  |  |  | X |  |
|  | PLA-6 |  |  |  | X |  |
|  | PLA-7 |  |  |  |  | X |
|  | PLA-8 |  |  |  |  | X |
|  | PLA-9 |  |  |  |  | X |
|  | PLA-10 |  |  |  |  | X |
|  | PLA-11 |  |  |  | X |  |
|  | PLA-12 |  |  | X |  |  |
| PLA-TQ | PLA-TQ-1 |  |  |  | X |  |
|  | PLA-TQ-2 |  |  |  | X |  |
|  | PLA-TQ-3 |  | X |  |  |  |
|  | PLA-TQ-4 |  |  | X |  |  |
|  | PLA-TQ-5 |  |  |  | X |  |
|  | PLA-TQ-6 |  |  | X |  |  |
|  | PLA-TQ-7 |  |  |  |  | X |
|  | PLA-TQ-8 |  |  |  |  | X |
|  | PLA-TQ-9 |  |  |  | X |  |
|  | PLA-TQ-10 |  |  | X |  |  |
|  | PLA-TQ-11 |  |  | X |  |  |
|  | PLA-TQ-12 |  |  |  | X |  |

*Macroscopic evaluation of intraabdominal adhesions using Modified Diamond Scale in study groups

**Histopathological Evaluation of Control Group**

| Histopathologic Parameters | | | | | | | |
| --- | --- | --- | --- | --- | --- | --- | --- |
| Granuloma | Lymphocyte infiltration | PMNL infiltration | Histiocytes | Giant cells | Capillary proliferation | Collagen content | Fibroblast proliferation |
| - | 3 | 2 | 2 | + | 3 | 2 | 2 |
| - | 2 | 3 | 3 | - | 2 | 3 | 2 |
| + | 3 | 3 | 2 | + | 3 | 2 | 3 |
| - | 2 | 2 | 2 | + | 2 | 2 | 2 |
| - | 3 | 2 | 2 | - | 2 | 1 | 2 |
| - | 2 | 3 | 2 | + | 2 | 3 | 2 |
| + | 3 | 4 | 3 | + | 2 | 3 | 3 |
| + | 3 | 3 | 2 | + | 2 | 2 | 2 |
| - | 2 | 3 | 2 | + | 2 | 2 | 2 |
| - | 2 | 3 | 2 | + | 3 | 2 | 2 |
| + | 2 | 3 | 3 | + | 3 | 2 | 2 |
| + | 2 | 3 | 2 | + | 2 | 2 | 3 |

**Histopathological Evaluation of PLA Group**

| Histopathologic Parameters | | | | | | | |
| --- | --- | --- | --- | --- | --- | --- | --- |
| Granulom | Lymphocyte infiltration | PMNL infiltration | Histiocytes | Giant cells | Capillary proliferation | Collagen content | Fibroblast proliferation |
| - | 2 | 2 | 2 | + | 3 | 2 | 2 |
| + | 2 | 3 | 4 | + | 2 | 2 | 3 |
| + | 2 | 3 | 1 | + | 2 | 3 | 3 |
| - | 2 | 2 | 1 | + | 1 | 2 | 2 |
| - | 2 | 2 | 2 | - | 2 | 2 | 2 |
| + | 2 | 3 | 2 | + | 3 | 2 | 2 |
| - | 3 | 2 | 2 | + | 3 | 2 | 2 |
| - | 2 | 2 | 1 | + | 2 | 2 | 1 |
| - | 2 | 2 | 2 | - | 2 | 3 | 3 |
| - | 2 | 3 | 1 | - | 3 | 3 | 2 |
| - | 1 | 2 | 1 | - | 2 | 3 | 2 |
| - | 3 | 2 | 1 | + | 2 | 2 | 2 |

**Histopathological Evaluation of PLA-TQ Group**

| Histopathologic Parameters | | | | | | | |
| --- | --- | --- | --- | --- | --- | --- | --- |
| Lymphocyte infiltration | PMNL infiltration | Histiocytes | Giant cells | Capillary proliferation | Collagen content | Fibroblast proliferation | Lymphocyte infiltration |
| - | 1 | 2 | 1 | - | 1 | 2 | 2 |
| - | 1 | 1 | 1 | - | 2 | 1 | 1 |
| - | 0 | 0 | 0 | - | 1 | 0 | 1 |
| - | 1 | 0 | 1 | - | 2 | 1 | 0 |
| - | 1 | 1 | 1 | - | 1 | 1 | 1 |
| - | 0 | 1 | 1 | - | 2 | 0 | 1 |
| - | 1 | 2 | 2 | - | 2 | 1 | 2 |
| - | 2 | 2 | 1 | - | 1 | 1 | 1 |
| - | 2 | 1 | 2 | - | 2 | 1 | 1 |
| - | 2 | 1 | 1 | - | 1 | 0 | 1 |
| - | 2 | 2 | 1 | - | 1 | 1 | 0 |
| - | 1 | 1 | 1 | - | 1 | 1 | 1 |

**Comparison of Mean, Standard Deviation, Median, Minimum, Maximum Values and p Values of all groups**

| Parameter | Group | # | Mean | SD | Mean | Min. | Max. | p value |
| --- | --- | --- | --- | --- | --- | --- | --- | --- |
| Granüloma formation | Control | 12 | 23,00 | ,522 | ,50 | 0 | 1 | 0,000 |
|  | PLA | 12 | 18,50 | ,452 | ,00 | 0 | 1 |  |
|  | PLA-TQ | 12 | 14,00 | ,000 | ,00 | 0 | 0 |  |
| Lymphocyte infiltration | Control | 12 | 25,13 | ,515 | 2,00 | 2 | 3 | 0,000 |
|  | PLA | 12 | 20,63 | ,515 | 2,00 | 1 | 3 |  |
|  | PLA-TQ | 12 | 9,75 | ,718 | 1,00 | 0 | 2 |  |
| PMNL infiltration | Control | 12 | 26,67 | ,577 | 3,00 | 2 | 4 | 0,000 |
|  | PLA | 12 | 20,50 | ,492 | 2,00 | 2 | 3 |  |
|  | PLA-TQ | 12 | 8,33 | ,718 | 1,00 | 0 | 2 |  |
| Histiocytes | Control | 12 | 26,88 | ,452 | 2,00 | 2 | 3 | 0,000 |
|  | PLA | 12 | 17,71 | ,888 | 1,50 | 1 | 4 |  |
|  | PLA-TQ | 12 | 10,92 | ,515 | 1,00 | 0 | 2 |  |
| Giant cell formation | Control | 12 | 24,50 | ,389 | 1,00 | 0 | 1 | 0,000 |
|  | PLA | 12 | 21,50 | ,492 | 1,00 | 0 | 1 |  |
|  | PLA-TQ | 12 | 9,50 | ,000 | ,00 | 0 | 0 |  |
| Capillary proliferation | Control | 12 | 23,17 | ,492 | 2,00 | 2 | 3 | 0,000 |
|  | PLA | 12 | 22,00 | ,622 | 2,00 | 1 | 3 |  |
|  | PLA-TQ | 12 | 10,33 | ,515 | 1,00 | 1 | 2 |  |
| Collagen content | Control | 12 | 22,92 | ,577 | 2,00 | 1 | 3 | 0,000 |
|  | PLA | 12 | 25,00 | ,492 | 2,00 | 2 | 3 |  |
|  | PLA-TQ | 12 | 7,58 | ,577 | 1,00 | 0 | 2 |  |
| Fibroblast formation | Control | 12 | 24,13 | ,452 | 2,00 | 2 | 3 | 0,000 |
|  | PLA | 12 | 22,96 | ,577 | 2,00 | 1 | 3 |  |
|  | PLA-TQ | 12 | 8,42 | ,603 | 1,00 | 0 | 2 |  |
| Modified Diamond Adhesion Score | Control | 12 | 20,75 | ,778 | 3,50 | 2 | 4 | 0,067 |
|  | PLA | 12 | 21,63 | ,669 | 3,50 | 2 | 4 |  |
|  | PLA-TQ | 12 | 13,13 | ,888 | 3,00 | 1 | 4 |  |
